# Trying to disentangle the causes of dyspnea and exercise limitation in a subject with GATA2 deficiency and normal spirometry: a case report

**DOI:** 10.3389/fphys.2026.1846805

**Published:** 2026-09-03

**Authors:** Giovanni Barisione, Carmela Di Grazia, Anna Maria Raiola, Emanuele Angelucci, Fabio Vignera, Vito Brusasco

**Affiliations:** 1 Struttura Semplice Fisiopatologia Respiratoria, Clinica Malattie Respiratorie e Allergologia, Istituto di Ricovero e Cura a Carattere Scientifico (IRCCS) Azienda Ospedaliera Metropolitana, Genova, Italy; 2 U.O. Ematologia e Terapie Cellulari, IRCCS Azienda Ospedaliera Metropolitana, Genova, Italy; 3 Dipartimento di Medicina Clinica e Sperimentale, Azienda Ospedaliero Universitaria Policlinico “G.Rodolico – San Marco”, Catania, Italy; 4 Dipartimento di Medicina Sperimentale, Università di Genova, Genova, Italy

**Keywords:** anemia, case report, dyspnea, emphysema, exercise limitation, GATA2 deficiency, Pneumocystis jirovecii, pulmonary diffusion

## Abstract

**Background:**

GATA-binding factor 2 (GATA2) deficiency is a genetic disorder characterized by prominent infectious and noninfectious pulmonary manifestations. Even though most patients with GATA2 deficiency seek medical attention for dyspnea and exercise intolerance, lung function data for this syndrome are scarce and rather inconclusive.

**Objective:**

To provide a comprehensive examination of lung function and quantitative computed tomography (CT) data and to assess responses to incremental exercise in a 38.5-year-old man with GATA2 deficiency and myelodysplastic syndrome, breathlessness, and normal spirometry.

**Methods:**

Lung volumes, spirometry, and single breath lung diffusing capacities for carbon monoxide (DL_CO_) and nitric oxide (DL_NO_) were sequentially measured. Subsequently, mean expired and arterial partial pressures of O_2_ and CO_2_ were obtained. The subject then performed a continuous (ramp-pattern) incremental [25 watts (W)·min^–1^] exercise test to a symptom-limited maximum on an electronically braked cycle ergometer.

**Results:**

Dyspnea and exercise intolerance were associated with *1)* anemia, *2)* severe impairment of alveolar-to-capillary gas diffusion with CT changes characterized by diffuse paraseptal emphysema and interstitial lung abnormalities associated with active Pneumocystis jirovecii infection, and *3)* abnormal responses to incremental exercise mainly due to a combination of mechanisms including decreased pulmonary gas exchange, anemia resulting in reduced blood O_2_-carrying capacity, metabolic acidosis, increased ventilatory drive, and deconditioning.

**Conclusions:**

This case study illustrates how clinical medicine benefits from applying the basic principles of respiratory physiology both at rest and during exercise to discern the mechanisms of dyspnea and exercise intolerance.

## 1 Introduction

GATA-binding factor 2 (GATA2) deficiency is a rare genetic disorder of hematopoiesis, lymphatics, and immunity caused by autosomal dominant or sporadic mutations in nuclear transcription GATA-binding factor 2 (Vinh et al., 2010). This syndrome encompasses a broad spectrum of conditions, including immunodeficiency, myelodysplastic syndrome (MDS), leukemia and vascular or lymphatic dysfunction in addition to prominent pulmonary manifestations. The latter include viral, fungal, and mycobacterial infections complicated in a minority (8%) of cases by pulmonary alveolar proteinosis (PAP) and/or pulmonary arterial hypertension (PAH) (Marciano et al., 2021; Vinh et al., 2010). Computed tomography (CT) of the chest may show small nodules in more than half (54%) if the subjects, whereas reticular infiltrates, ground-glass opacities, and bronchiectases were found in 40%, 36% and 10% of them, respectively. Moreover, one-fourth (25%) of subjects had paraseptal emphysema (Marciano et al., 2021). For these reasons, most subjects with GATA2 deficiency seek medical attention for exercise intolerance, fatigue, breathlessness, and cough. The histopathological and radiological features of GATA2 deficiency have been well described, whereas lung function data are rather inconclusive (Marciano et al., 2021). A reduction in lung diffusing capacity for carbon monoxide (DL_CO_) was reported in the majority of cases, while variable spirometric abnormalities were present in less than half. However, in the absence of measurements of total lung capacity (TLC) the precise nature of spirometric abnormalities may be difficult to determine (Stanojevic et al., 2022). In a minority of cases, a reduction in 6-min walking distance was also reported, but this is a measure of functional capacity that cannot provide information on the mechanisms of exercise limitation (Baccelli et al., 2025). For these reasons, the pathophysiology of breathlessness and exercise intolerance in GATA2 deficiency remains largely unexplained.

The purpose of this article is to provide a comprehensive examination of lung function and quantitative CT data in a case of GATA2 deficiency associated with MDS. Moreover, by measuring the response to exercise, we aim to examine the underlying pathophysiological mechanisms of dyspnea and exercise limitation that, singly, but more commonly in combination, may limit or prevent the performance of daily activities by subjects with this condition.

## 2 Materials and methods

### 2.1 Participant and case description

**Figure 1:**
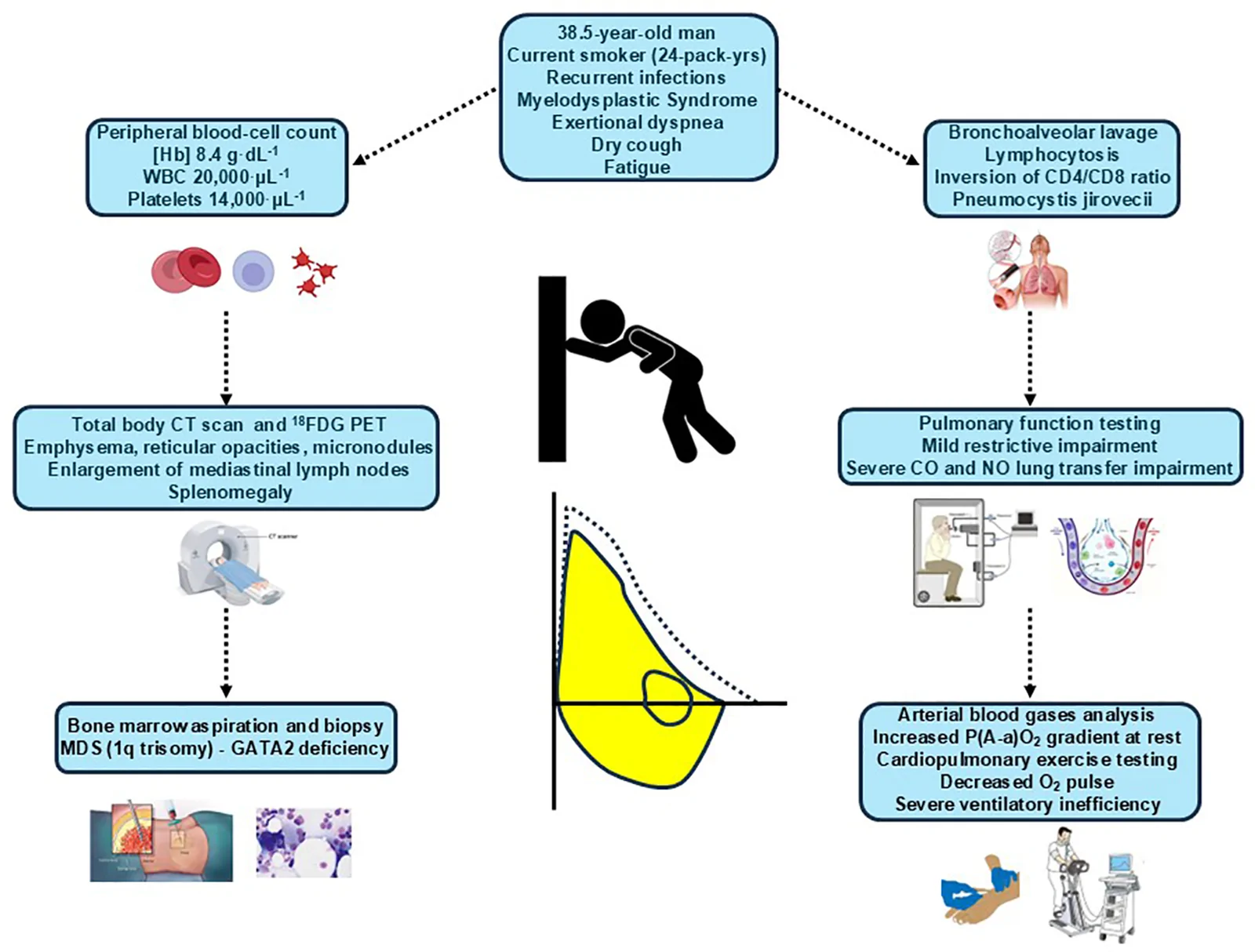
Clinical events, laboratory studies, imaging procedures, and lung function measurements in a subject with GATA2 deficiency and myelodysplastic syndrome. Figure partially created with BioRender.com, with permission.

Data are presented for a 38.5-year-old man of European descent with normal body mass index (BMI: 25 kg·m^-2^), currently a smoker (24-pack-years), a ceramic worker who was referred to our institution by a regional hospital. In 2006, he was diagnosed with MDS without blasts, and 8 years later, he was prescribed INN-epoetin-α owing to progressive anemia. Because of recurrent respiratory infections, diffuse lymph node enlargement, and splenomegaly, he was subsequently evaluated in 2024 for allogeneic hematopoietic stem-cell transplantation (HSCT). In 2025, on admission as an outpatient, he experienced exertional dyspnea, dry cough, and fatigue (**Figure 1**). A peripheral blood cell count showed 2,900,000 red blood cells·μL^–1^ with a hemoglobin concentration ([Hb]) of 8.4 g·dL^–1^, 21,100 white blood cells·μL^–1^, and 14,000 platelets·μL^–1^. A transthoracic Doppler echocardiography revealed a normal ejection fraction (~65%) and non-titratable pulmonary artery systolic pressure. CT of the chest revealed moderate paraseptal and centrilobular emphysema, micronodules, “*ground glass*” opacities and interlobular reticular opacities with traction bronchiectases. A positron emission tomography confirmed significant 18-fluorodeoxyglucose uptake (SUVmax ~7) in these lesions. Flow cytometry showed low monocytes/B/NK cells and inversion of CD4/CD8 ratio, whereas peripheral blood leukocyte DNA next-generation sequencing identified a heterozygous autosomal dominant inactivating variant (c.787G>T) of the GATA2 gene. A few days later, a fiber optic bronchoscopy with examination of bronchoalveolar lavage fluid (BALF) revealed lymphocytosis with an inverted CD4/CD8 ratio. Because of the isolation of Pneumocystis jirovecii (P. jirovecii) in BALF, trimethoprim-sulfamethoxazole (20 mg·kg^–1^ and 100 mg·kg^–1^ daily, respectively) was administered. In December 2025, he underwent a matched unrelated donor allogeneic HSCT and is currently being followed up as an outpatient.

### 2.2 Lung function procedures and measures

The subject visited the pulmonary function tests (PFTs) laboratory, located 160 m above sea level, on two separate occasions. Prior to arrival, he was instructed to refrain from smoking 24 h before the measurements, and smoking abstinence was confirmed before testing. At the beginning of the first session, the modified Medical Research Council (mMRC) Dyspnea Scale (grades 0-4) was used to measure the level of functional disability caused by breathlessness. Subsequently, lung volumes (Bhakta et al., 2023), spirometry (Graham et al., 2019), and single breath DL_CO_ (Graham et al., 2017) with 10.7 seconds actual breath-hold time (BHT), adjusted for effective [Hb] (Cotes et al., 1972), were sequentially measured with the subject wearing a nose clip and sitting in a whole-body plethysmograph (Vyntus™ BODY Plethysmograph, Vyaire Medical, Höchberg, Germany). The prediction equations for all standard lung function parameters were obtained from the Global Lung Function Initiative (Hall et al., 2021; Quanjer et al., 2012; Stanojevic et al., 2017). Subsequently, the single-breath lung diffusing capacity for nitric oxide (DL_NO_), with 5.2 seconds BHT, was measured (MasterScreen-PFT System; Jaeger, Vyaire Medical, Höchberg, Germany) twice at a 5-min interval and the values retained for analysis were the average of two repeatable measurements, *i.e.*, measurements with values within 17.0 mL·min^–1^·mmHg^–1^ of each other (Zavorsky et al., 2017). The prediction equations for DL_NO_ were obtained from Munkholm et al (Munkholm et al., 2018). Lung function data were interpreted according to the European Respiratory Society/American Thoracic Society technical standard, and the 5^th^ and 95^th^ percentile limits (-1.645 and 1.645 *z*-scores, respectively) of the healthy population were used to identify abnormally low or high results, respectively (Stanojevic et al., 2022).

### 2.3 Resting arterial blood gases analysis

Nineteen days after the first visit, exhaled O_2_ and CO_2_ concentrations were measured at the mouth and averaged at rest over 10 tidal-volume (V_T_) breaths while the subject was seated upright, wearing a nose clip, and breathing ambient air through a flanged mouthpiece (Vyntus™ CPX Metabolic Cart, Vyaire Medical, Höchberg, Germany) to determine mean expired O_2_ and CO_2_ partial pressures ( $P_{\bar{E}}O_{2}$ and $P_{\bar{E}}CO_{2}$, respectively). The right radial artery was simultaneously punctured, and aspiration was intentionally prolonged over at least 2–4 tidal volume (V_T_) breaths to obtain a single blood sample (~1 mL) over several breaths. The sample was analyzed within 15–30 seconds for mean arterial O_2_ and CO_2_ partial pressures ( $PaO_{2}\;$and $PaCO_{2}$, respectively) (Blood Gas Analyzer ABL90 Flex Radiometer, Copenhagen, Denmark). To avoid potential bias due to the inappropriate assumption of $PACO_{2}\approx PaCO_{2}$ in the alveolar air equation, the alveolar-to-arterial PO_2_ $[P(A-a)O_{2}$] gradient was obtained using a comparison of $P_{\bar{E}}O_{2}$ with $PaO_{2}$ (Wagner et al., 2022). The physiological dead space ( $V_{D}$)-to- $V_{T}$ ratio was calculated using Enghoff’s modified Bohr equation (Enghoff, 1938):


$$\frac{{\text{V}}_{\text{D}}}{{\text{V}}_{\text{T}}}=(\frac{\text{PaC}{\text{O}}_{2}-{\text{P}}_{\bar{\text{E}}}\text{C}{\text{O}}_{2}}{\text{PaC}{\text{O}}_{2}})$$

The prediction equation for $PaO_{2}$ was obtained from Hughes (Hughes, 2007) whereas $P(A-a)O_{2}\;$and V_D_/V_T_ were obtained from Gläser et al (Gläser et al., 2013). Finally, the O_2_-carrying capacity (content) of arterial blood ( $Ca_{O_{2}}$) was calculated as follows (Finch and Lenfant, 1972):


$$\text{Ca}{\text{O}}_{2}=(\text{Pa}{\text{O}}_{2}\cdot \text{βp}{\text{O}}_{2})+(\text{Sa}{\text{O}}_{2}\cdot [\text{Hb}]\cdot 1.31)$$

where $\beta pO_{2}$ is the capacitance coefficient (physical solubility) of O_2_ in plasma at 37 °C (~0.003 mL·dL^–1^·mmHg^–1^), $SaO_{2}$ is the fractional O_2_ saturation of arterial blood, and 1.31 mL O_2_·g [Hb]^–1^ corresponds to the value of Hüfner’s constant *in vivo* (Lumb, 2000).

### 2.4 Incremental cardiopulmonary exercise testing

After measurements of resting spirometry and 12-s maximum voluntary ventilation (MVV), the subject, in a sitting position, with nose clip and mouthpiece in place, began 3 min of unloaded pedaling and then performed a continuous (ramp-pattern) incremental [25 watts (W)·min^-1^] exercise test to a symptom-limited maximum on an electronically braked cycle ergometer (XR100 Cicloergometro, Cardioline S.p.A.; Spini di Gardolo, TN, Italy), maintaining a cycling speed of ~60 rpm. The rate of work increase (25 W·min^-1^) was selected as follows:

peak oxygen uptake ( $\dot{\text{V}}{\text{O}}_{2}$) (mL·min^-1^) - unloaded $\dot{\text{V}}{\text{O}}_{2}$ (mL·min^-1^)/100 (Wasserman et al., 2012).

Heart rate (HR), respiratory frequency ( $f_{R}$), $V_{T}$, expiratory minute ventilation ( $\dot{V}E$), $\dot{V}O_{2}$, carbon dioxide production ( $\dot{V}CO_{2}$), and their ratios were recorded breath-by-breath throughout the test (Vyntus™ CPX Metabolic Cart, Vyaire Medical, Höchberg, Germany). Maximum predicted HR was calculated using the equations reported in Tanaka et al (Tanaka et al., 2001). Prediction equations for maximum values of work rate (WR), $\dot{V}O_{2}$, $\dot{V}E$, $\;\dot{V}CO_{2}/\dot{V}O_{2},\;$*i.e.*, respiratory exchange ratio (RER) and $\dot{V}E$ *vs.* $\dot{V}CO_{2}\;$slopes were obtained from Gläser et al (Gläser et al., 2013). Maximum values during incremental cycle ergometry were confirmed by attainment of a plateau, *i.e.*, an increment <2.1 mL·kg^–1^·min^–1^, in $\dot{V}O_{2max}$ (Taylor et al., 1955) and an RER >1.645 *z*-score (>95^th^ percentile) (Murgatroyd et al., 2014). Earlobe pulse oximetry (SpO_2_) (Xpod^®^ 3011LP; PureSAT|SpO_2_, Nonin, Plymouth, MN, USA), a continuous 10-lead electrocardiogram (ECG) and automated brachial arterial blood pressure (Tango M2 Stress Test Monitor, SunTech Medical, Morrisville, NC, USA) were recorded. Rating of perceived exertion (RPE) or magnitude of dyspnea experienced during exercise was recorded immediately after completion of CPET (Borg, 1982). For arterial blood and CPET measures, the 5^th^ and 95^th^ percentile limits (-1.645 and 1.645 *z*-scores) of the healthy population were used to identify abnormally low or high results, respectively.

### 2.5 Quantitative CT parenchymal and airway analysis

Volumetric CT scans of the chest (1.25-mm slice thickness, 200 mAs, 120 kVp) were obtained 7 days before PFTs, during a breath-hold at full inspiration, using a multi-detector row-spiral scanner (SOMATOM Emotion 6; Siemens AG Medical, Forchheim, Germany). The lungs were automatically segmented (Fedorov et al., 2012). A noise reduction filter was applied to decrease the influence of noise on the quantitative measurements. Within the segmented lung volume, the attenuation in Hounsfield Units (HU) of each voxel was assessed to quantify emphysema severity defined as the percentage of voxels in inspiratory CT with low attenuation areas <-950 HU (LAA_<-950 HU_) (Stoel et al., 2008; Lynch et al., 2015). To quantify the interstitial lung abnormalities, the percentage of voxels in inspiratory CT with high attenuation areas >-600 HU (HAA_>-600 HU_) was measured (Wang et al., 2024) (3D Slicer 5.6.1 software; https://www.slicer.org/) without manual correction. For quantification of airway wall thickness, the airway lumen and its centerline were determined in cross-sections perpendicular to the local airway dimension (3D Slicer 5.6.1 software; https://www.slicer.org/). The mean airway wall area-to-total bronchial cross- sectional area ratio (AW%) of the right and left subsegmental B1, B4 and B10 (3^rd^ Boyden’s generation) branches were measured (Dudurych et al., 2022).

## 3 Results

### 3.1 Lung function

The subject’s lung function data are shown in **Table 1**. Forced vital capacity (FVC), forced expiratory volume in 1 second (FEV_1_), and the FEV_1_/FVC ratio were slightly above the lower limit of normal (-1.645 *z*-score) at 5^th^ percentile of reference population (LLN_5_) whereas TLC was slightly below (-1.860 *z*-score). Most notably, DL_CO_, DL_NO_ and their ratios to alveolar volume (V_A_) were severely decreased. Additional forced expiratory flows and lung volumes data are reported in **Supplementary Table 1**.

**Table 1:** Subject’s lung function data.

Resting lung function parameters	Absolute, % of Predicted and z-score values
FVC, L % of Predicted *z*-score	4.05 84 -1.282
FEV_1_, L % of Predicted *z*-score	3.14 80 -1.572
FEV_1_/FVC *z*-score	0.78 -0.669
TLC, L % of Predicted *z*-score	5.27 80 -1.670
RV, L % of Predicted *z*-score	1.22 66 -1.530
Hb, g·dL^-1^	8.4
DL_CO_, mL·min^-1^·mmHg^-1^ % of Predicted *z*-score	13.99 48 -4.428
K_CO_, mL·min^-1^·mmHg^-1^·L^-1^ % of Predicted *z*-score	2.64 55 -3.733
DL_NO_, mL·min^-1^·mmHg^-1^ % of Predicted *z*-score	71.1 50 -4.249
K_NO_, mL·min^-1^·mmHg^-1^·L^-1^ % of Predicted *z*-score	13.4 50 -6.559

FEV_1_, forced expiratory volume in 1 s; FVC, forced vital capacity; DL_CO,_ single-breath lung diffusing capacity for carbon monoxide, adjusted for [Hb], with an 11.7-s BHT; DL_NO,_ single-breath lung diffusing capacity for nitric oxide with a 5.2-s BHT; K_CO,_ DL_CO_/alveolar volume; K_NO,_ DL_NO_/alveolar volume; RV, residual volume; TLC, total lung capacity.

### 3.2 Resting arterial blood measures

$PaO_{2},\;PaCO_{2}\;$and $[HCO_{3}^{-}$] values were significantly decreased, indicating mild arterial hypoxemia (78.5 mmHg; -2.071 *z*-score) and hypocapnia (30.6 mmHg) associated with compensatory hyperventilation ( ${\dot{V}}_{E}$ 15.8 ± 6.94 L·min^-1^) at rest (**Table 2**). Moreover, $P(A-a)O_{2}$ was increased (48.8 mmHg; 2.957 *z*-score) indicating a moderate pulmonary gas-exchange inefficiency associated with an increased V_D_/V_T_ ratio (0.44; 3.138 *z*-score). Because of the combination of severe anemia ([Hb] 8.4 g·dL^–1^) and mild hypoxemia, $Ca_{O_{2}}$ was severely decreased (10.9 mL·dL^–1^, -46% *vs.* a normal mean value of ~20.2 mL·dL^–1^).

**Table 2:** Resting arterial blood and exercise parameters and exercise measures.

Resting arterial blood and exercise parameters	Absolute and z-score values
$\dot{V}E_{rest}$, L·m^–1^	15.8 ± 6.94
pHa	7.428
$PaO_{2}$, mmHg *z*-score	78.5 -2.071
$PaCO_{2}$, mmHg	30.6
$SaO_{2}$, %	97.1
$[HCO_{3}^{-}$], mM·L^-1^	20.2
$P(A-a)O_{2}$, mmHg *z*-score	48.8 2.957
$CaO_{2}$, mL·dL^–1^	10.9
Lactate, mM·L^–1^	1.0
HbCO, %	2.4
$WR_{max}$, W *z*-score	98 -2.732
$\dot{V}O_{2max}$, mL·min^–1^ *z*-score	1098 -3.256
$\dot{V}O_{2max}$/kg, mL·min^–1^·kg^–1^	15.3
$\dot{V}O_{2}@AT$, mL·min^–1^ *z*-score	768 -1.894
$\Delta \dot{V}O_{2}/\Delta WR$, mL·min^–1^·W^–1^	6.92
$\dot{V}CO_{2max}$, mL·min^-1^	1446
RER_max_ *z*-score	1.32 2.354
$\dot{V}E\;$*vs.* $\dot{V}CO_{2}$ slope *z*-score	39.4 4.596
$\dot{V}E_{max}/MVV$	0.68
$\dot{V}O_{2max}/HR$_max_ , mL·beat^–1^ *z*-score	7.50 -2.989
$SpO_{2max}$, %	96
RPE dyspnea (Borg)	5/10
RPE leg discomfort (Borg)	8/10

AT, anaerobic threshold; $CaO_{2}$, arterial O_2_ content or carrying capacity; $[HCO_{3}^{-}$], arterial standard bicarbonate ion concentration; HR, heart rate; MVV = 12-s maximum voluntary ventilation; $PaCO_{2}$, arterial carbon dioxide partial pressure; $PaO_{2}$, arterial oxygen partial pressure; $P(A-a)O_{2}$, alveolar-to-arterial O_2_ gradient; RER_max_, maximum respiratory exchange ratio, *i.e.*, $\dot{V}CO_{2}/\dot{V}O_{2}$; RPE, rate of perceived exertion; $SaO_{2}$, arterial oxyhemoglobin saturation; SpO_2_, pulse oximetry; $\dot{V}O_{2max}$, maximum O_2_ uptake per minute; $\dot{V}O_{2}@AT$, O_2_ uptake per minute at anaerobic threshold; $\Delta \dot{V}O_{2}/\Delta WR$, slope of $\dot{V}O_{2}$ as a function of work rate; $\dot{V}CO_{2max}$, maximum CO_2_ output per minute; $\dot{V}E_{max}$, maximum expiratory minute ventilation; $\dot{V}E_{rest}$, expiratory minute ventilation at rest; $WR_{max}$, maximum work rate.

### 3.3 Exercise performance and aerobic power

**Figure 2:**
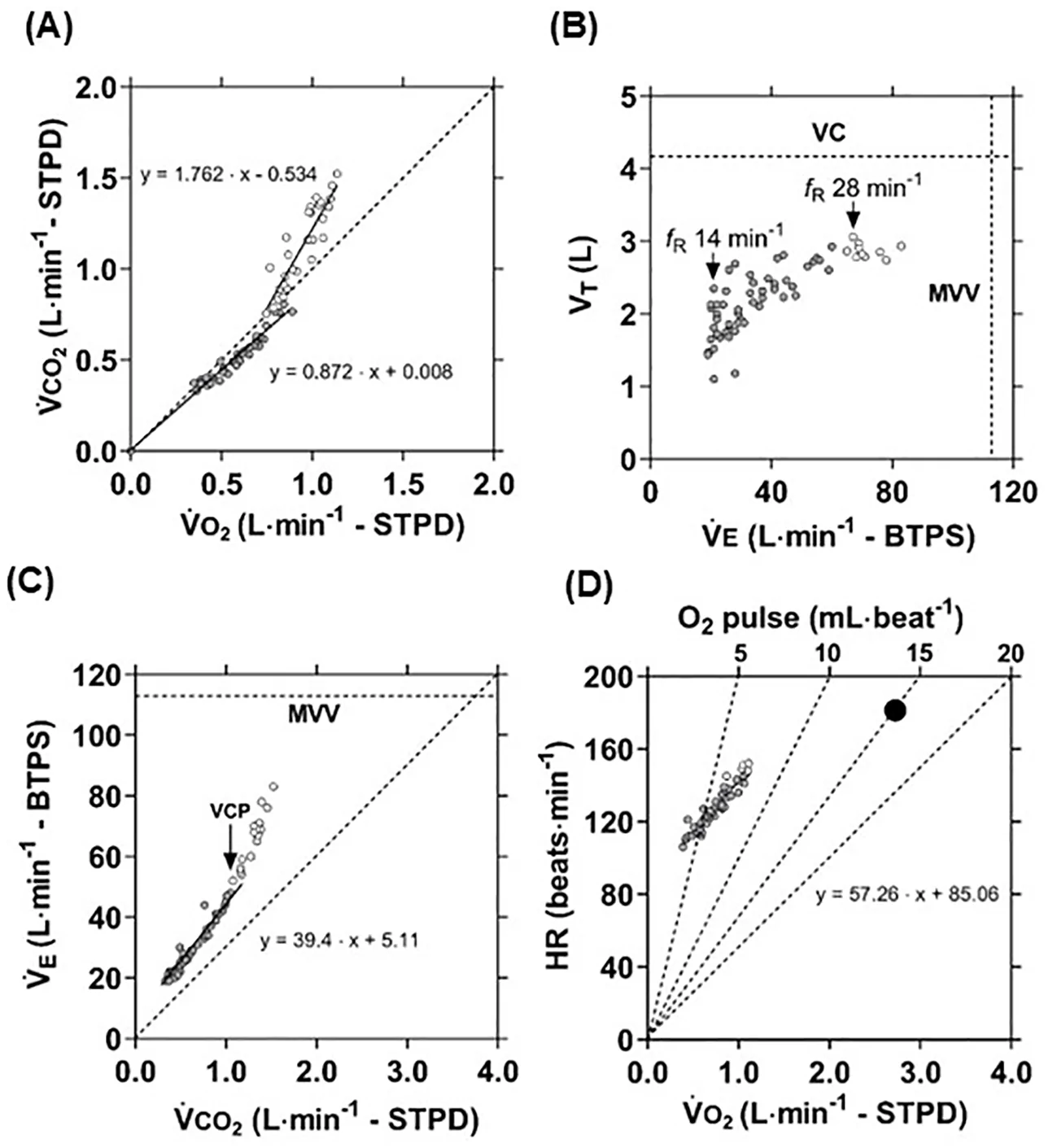
Values of O_2_ uptake ( $\dot{V}O_{2})$, CO_2_ output ( $\dot{V}CO_{2}$), expired minute ventilation $(\dot{V}E$), tidal volume (V_T_), O_2_ pulse and heart rate (HR) for the study subject during a progressively increasing (ramp) cycle ergometer test. For clarity, the resting, unloaded pedaling, and recovery data are not shown. Circles indicate the relevant parameter value before (*gray*) and after (*white*) anaerobic threshold or ventilatory compensation point (VCP). In **(A)**, the diagonal dashed line corresponds to the line of identity whereas in **(B, C)**, the horizontal and vertical dashed lines correspond to vital capacity (VC) or measured maximum voluntary ventilation (MVV). In **(D)**, isopleths for O_2_ pulse at 5, 10, 15, and 20 mL per beat are shown. The large solid (black) circle indicates the maximal exercise target values.

At rest, the subject had a grade 3 mMRC dyspnea score, while HR and SpO_2_ were 108 beats·min^–1^ and 99%, respectively. The subject pedaled with added load for 4:13 min, reaching a workload of 98 W (-2.732 *z*-score) and the Borg RPE scores were 8 for leg fatigue and 5 for breathlessness. The $\dot{V}O_{2max}$ was substantially decreased (1098 mL·min^–1^; -3.256 *z*-score) whereas external work efficiency ( $\Delta \dot{V}O_{2}/\Delta WR$ slope: 6.92 mL·min^-1^·W^-1^) and $\dot{V}O_{2}\;$at the anaerobic threshold $(\dot{V}O_{2}@AT$: 768 mL·min^-1^; -1.894 *z*-score) were mildly decreased (**Figure 2**).

### 3.4 Ventilatory response and gas exchange

$\dot{V}E\;$and *f*_R_ increased from 23 L·min^–1^ and 14 min^–1^ at rest to 76 L·min^–1^ and 28 min^–1^ at 98 W, respectively. The breathing reserve $\left(\dot{V}E_{max}-MVV\cdot 100\right)$ was ~36% whereas the ventilatory efficiency was severely impaired, as shown by the steep upward slope of $\dot{V}E\;$*vs.* $\dot{V}CO_{2}$ (39.4 with 5.11 *y*-intercept; 4.596 *z*-score for the former), before the ventilatory compensation point (VCP) associated with normal V_D_/V_T_ (0.22; *z*-score 0.138) and slightly decreased SpO_2_ (96%; -3% *vs.* the resting value).

### 3.5 Cardiovascular responses

At rest, the subject was in sinus rhythm, and HR increased to 152 beats·min^-1^ (with an HR reserve of ~29 beats·min^–1^) at 89 W. Interestingly, the peak O_2_ pulse was substantially decreased (7.50 mL·beat^–1^; -2.989 *z*-score) with a normal rate of rise of HR *vs.* $\dot{V}O_{2}$ but early exercise termination at a relatively low work rate. There were no ST-segment changes or arrhythmias on ECG.

### 3.6 Quantitative CT of the chest

**Figure 3:**
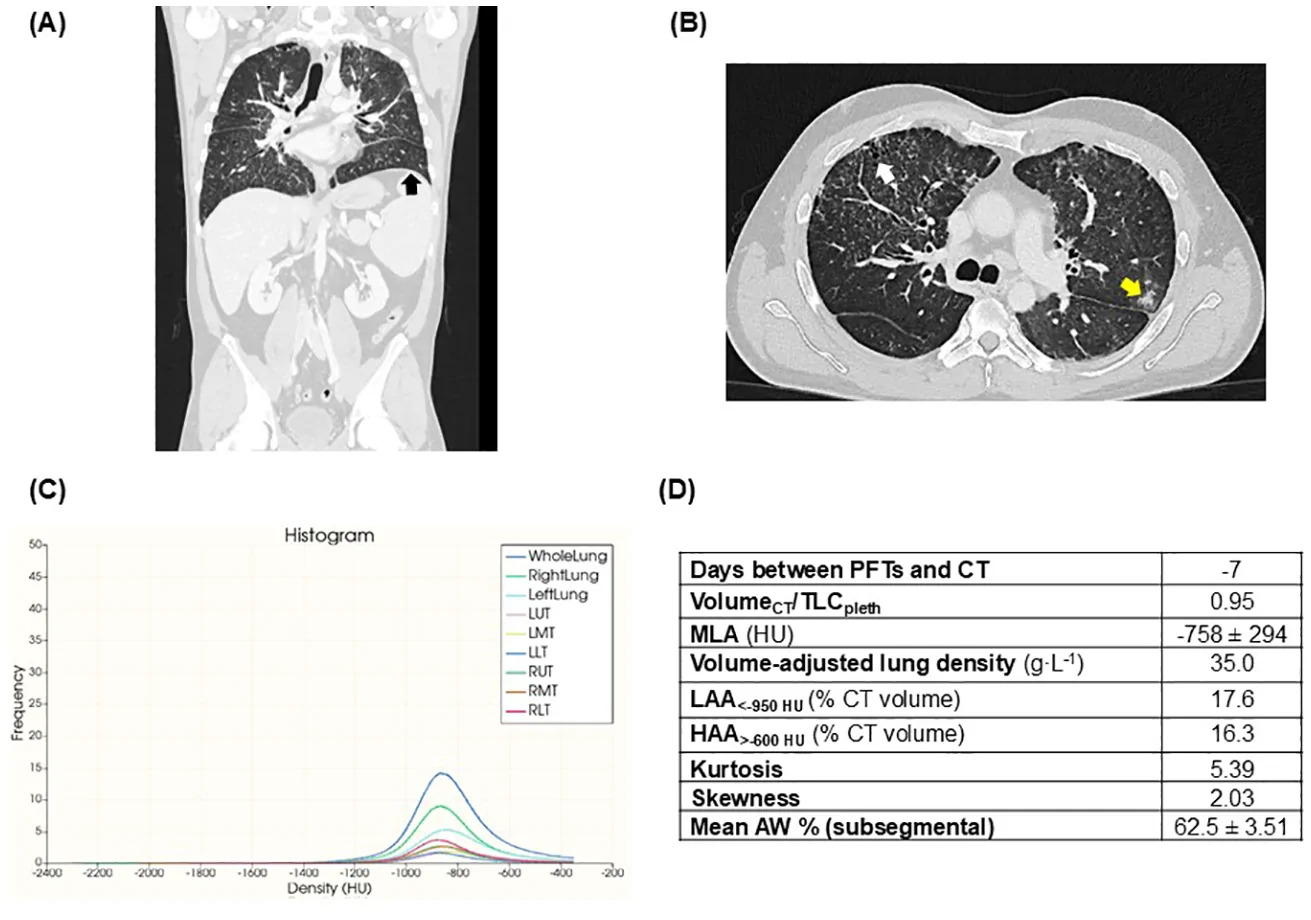
Total-body coronal **(A)** and chest axial [carinal **(B)**] CT scans of the study subject. Slight elevation of the left hemidiaphragm, probably due to splenomegaly (black arrow) is shown in **(A)**, while in **(B)**, subpleural paraseptal emphysema and a ground-glass opacity are indicated by white and yellow arrows, respectively. **(C, D)** display the lung density histogram and quantitative parenchymal and airway wall data.

Volume-adjusted lung density was severely decreased (35 g·L^-1^) and voxels with both LAA_<-950 HU_ and HAA_>-600 HU_ as a percentage of CT lung volume, indicating emphysema and interstitial lung abnormalities, respectively, were significantly increased (**Figure 3**). The AW% value of subsegmental bronchi was mildly increased.

## 4 Discussion

To our knowledge, this is the first comprehensive description of lung function data, quantitative CT changes, and exercise responses in an individual with GATA2 deficiency. The main findings of the present study are that breathlessness and exercise intolerance in our subject were multifactorial and associated with *1)* severe anemia, *2)* impairment of alveolar-to-capillary gas diffusion with CT-determined paraseptal emphysema and interstitial lung abnormalities associated with active P. jirovecii infection, and *3)* abnormal responses to incremental exercise with reductions in $\dot{V}O_{2}$ and O_2_ pulse, mainly due to a combination of mechanisms including impaired pulmonary gas exchange, anemia resulting in reduced blood O_2_-carrying capacity, metabolic acidosis, increased ventilatory drive, and deconditioning.

### 4.1 Comments on resting lung function data

In this syndrome, dyspnea, cough, and exercise intolerance are often associated with the pulmonary manifestations of the disease. The respiratory tract is frequently affected by viral, fungal, or mycobacterial infections, sometimes complicated by PAP and PAH (Marciano et al., 2021). On CT scans, reticular and ground-glass opacities, nodules, and diffuse paraseptal emphysema with subpleural blebs are common. In particular, paraseptal emphysema, representing peripheral destruction of subpleural parenchyma and its gas-exchanging acini, may be extensive and progress over time (Marciano et al., 2021). PFT data were available for a limited number of subjects and were characterized by a decreased DL_CO_ and variable spirometric abnormalities that were difficult to interpret because of bias introduced by the use of percentages of predicted values rather than percentile values and the unavailability of TLC (Marciano et al., 2021). Several studies have consistently shown that DL_CO_ may be reduced in hematological disorders either before (Afessa, 2005; Barisione et al, 2008) or after HSCT (Rodríguez-Roisin et al., 1989; Barisione et al, 2008; Barisione et al., 2014). Our subject showed a mild restrictive abnormality (TLC, -1.860 *z*-score) probably due to the elevation of the left hemidiaphragm associated with spleen enlargement. This pattern was associated with impairment of DL_CO_, DL_NO_, and $P(A-a)O_{2}$. Because DL_NO_ is weighted predominantly (~60%) toward alveolar membrane diffusive conductance, is deemed to be a more specific marker of alveolar-capillary destruction than DL_CO_ (Barisione et al., 2016, Barisione et al., 2019, Barisione et al., 2026). Moreover, due to its relative insensitivity to intrapulmonary hematocrit, DL_NO_ does not require adjustment for effective [Hb] in anemia (van der Lee et al., 2005). For this reason, we hypothesize that DL_CO_ and DL_NO_ impairment are mainly due to paraseptal and centrilobular emphysema, *i.e.*, increased LAA_<-950 HU,_ shown by quantitative chest CT and, to a lesser extent, interstitial lung abnormalities (increased HAA_>-600 HU_). However, lung function data and CT findings in our subject must be interpreted with a modicum of caution. Indeed, because he was a current smoker and developed emphysema as a consequence, interstitial lung abnormalities and reduced pulmonary diffusion may all exhibit features of smoking-related lung injury. Moreover, *P. jirovecii* attaches to type I pneumocytes, and active infection may be associated with diffuse bilateral interstitial infiltrates and impairment of pulmonary gas exchange (Selwyn et al., 1998).

### 4.2 Comments on exercise testing

A previous study showed a decreased 6-min walking distance in subjects with GATA2 deficiency (Marciano et al., 2021). However, even though it may be a simple test to assess functional capacity, this measure is of limited diagnostic value because maximum cardiac output and $\dot{V}O_{2max}$ are rarely achieved during walking in many patients (Oudiz et al., 2006).

Our subject performed a progressive, incremental (ramp) cycle ergometer exercise and stopped pedaling prematurely (98 W) at low $\;\dot{V}O_{2max}.$ The severe impairment of DL_CO_, DL_NO_ and $P(A-a)O_{2}$ at rest and the increased $\dot{V}E\;$*vs.* $\dot{V}CO_{2}\;$slope with a low, positive, *y*-intercept during exercise would support diffusion limitation, *i.e.*, a low $\frac{DL}{\beta \dot{Q}}$ratio, *i.e.*, the diffusion/perfusion ( $\dot{Q}$) conductance ratio, as an important determinant of exercise limitation (Scheid and Piiper, 1989). The resting DL_CO_ has been previously linked to a reduced ventilatory efficiency and the resultant elevated inspiratory neural drive to the respiratory muscles during exercise, measured by diaphragm electromyography (James et al., 2021). However, the equilibration deficit [ $P(A-a)O_{2}$]) relative to the entrance difference $\;[P(A-\bar{v})O_{2}$], exacerbated by the shortening of the mean transit time of capillary blood, does not fully explain our findings because our subject showed only a mild (-3%) decrease in SpO_2_ compared to the resting state. On the other hand, the normal V_D_/V_T_ ratio (0.22) before VCP observed lends support to the recruitment-dilation of pulmonary vessels, which improve perfusion in high alveolar ventilation/perfusion ( ${\dot{V}}_{A}$/ $\dot{Q}$) areas that were likely present at rest. An additional and, perhaps, more important explanation for exercise limitation in this subject may be the severe anemia (8.4 g·dL^-1^ [Hb]) and decreased (~50%) $CaO_{2}$ that compromises O_2_ delivery to the mitochondria. The role of anemia in the reduction of $\dot{V}O_{2}$ can be calculated in normoxia (Wasserman et al., 2012). Although our subject was slightly hypoxemic (PaO_2_ 78.5 mmHg; 84% predicted) at rest, he showed SpO_2_ values of 96% at peak exercise. Thus, considering a peak cardiac output ( ${\dot{Q}}_{T})$, calculated by Fick’s principle, of ~4.03 L·min^–1^, it is reasonable to assume that, with an O_2_ extraction rate at peak exercise of ~75%, each gram of Hb would deliver ~0.98 mL of O_2_. Hence, 8.4 g·dL^–1^ [Hb] accounted for a $\dot{V}O_{2}$ value of 338.5 mL·min^–1^ instead of the expected 588.4 mL·min^–1^ (-42%) calculated using a normal [Hb] of 14.6 g·dL^–1^. Consequently, a critically low capillary PO_2_ may be reached at a lower-than-normal $\dot{V}O_{2}$, necessitating anaerobic mechanisms for ATP regeneration, with increasing lactate concentrations, metabolic acidosis, and increased $\dot{V}E\;$*vs.* $\dot{V}CO_{2}\;$slope due to inappropriate ventilatory drive (Wasserman et al., 2012). This mechanism is supported by the low $\dot{V}O_{2}@AT$ observed and, in particular, the substantial decrease in O_2_ pulse. The latter response is most commonly due to a low stroke volume, but this is not the case in our subject because he had normal echocardiographically-determined ejection fraction and serum NT-pro-brain natriuretic peptide (70 ng·L^–1^) at rest. Thus, the low O_2_ pulse is probably due to a combination of anemia, poor blood oxygenation in the lungs and low peripheral O_2_ extraction. For these reasons, the rate of increase in HR *vs.* $\dot{V}O_{2}$ is normal, but exercise ends at a relatively low work rate.

### 4.3 Study limitations

*First*, we did not measure changes in arterial blood gases, pH, [ $HCO_{3}^{-}$] and lactate during exercise. However, our subject’s platelet count was severely decreased, and radial artery catheterization could have been at risk of undue hemorrhagic complications. Moreover, the blood lactate value was normal at rest (1.0 mM·L^–1^) whereas exercise-induced lactic acidosis was supported by the marked increase in the slope (1.762) at the breaking point of $\dot{V}CO_{2}\;$*vs.* $\dot{V}O_{2}$ regression (Beaver et al., 1986) and the high RPE (8/10) for leg fatigue. *Second*, patients with hematological disorders may have increased carboxy-hemoglobin levels(>3%), as a byproduct of enhanced heme catabolism, with a leftward shift of the O_2_ dissociation curve that may contribute to reduced O_2_ transport to tissues. However, this was not the case in our subject, since he showed a normal value (2.4%) of carboxyhemoglobin. *Third*, we did not titrate autoantibodies against granulocyte macrophage colony-stimulating factor in the serum and/or BALF of this subject (Bonfield et al., 2002). However, owing to the low CD4+/CD8+ ratio found in BALF, we deemed that secondary alveolar proteinosis was unlikely in this subject.

## 5 Conclusions

Discerning the causes of dyspnea in a complex disease such as GATA2 deficiency may be a daunting task. Taken together, the data from this study suggest that a combination of central and peripheral mechanisms, including anemia, impaired pulmonary diffusion, inefficient gas exchange, and decreased O_2_ delivery and extraction, with low work rate lactic acidosis, and deconditioning, may play a role. These pathophysiological changes can occur singly, but more commonly they occur in combination. This case study highlights that applying the basic principles of respiratory physiology to clinical findings may help to discern the mechanisms of dyspnea and exercise intolerance.

## Data Availability

The original contributions presented in the study are included in the article/**Supplementary Material**. Further inquiries can be directed to the corresponding author.
