# Supplementary material for: Trying to disentangle the causes of dyspnea and exercise limitation in a subject with GATA2 deficiency and normal spirometry: a case report
Source: Front Physiol. 2026 Sep 3;17:1846805. doi: 10.3389/fphys.2026.1846805 (PMC13581732; doi:10.3389/fphys.2026.1846805)
Supplement: Supplementary file 2 [file DataSheet2.pdf]

Supplemental Table S1. Subject's forced expiratory flows and lung volumes

**PEF**, $L \cdot s^{-1}$	7.86
$\dot{\mathbf{V}}\mathbf{E}_{\mathbf{max\ 25\%}}$, $L \cdot s^{-1}$	0.90
$\mathbf{FEF_{25\text{-}75\%}}$, $L \cdot s^{-1}$<br>% of Predicted<br>*z*-score	2.71<br>69<br>-1.190
**VC**, L<br>% of Predicted<br>*z*-score	4.05<br>86<br>-1.170
$\mathbf{V_T}$, L	0.95
**IRV**, L	2.04
**ERV**, L<br>% of Predicted<br>*z*-score	1.06<br>75<br>-0.687

Definition of abbreviations: ERV = expiratory reserve volume; $FEF_{25\text{-}75\%}$: forced mid-expiratory flow between 25% and 75% of FVC; IRV = inspiratory reserve volume; PEF = peak expiratory flow; $\dot{V}E_{max\ 25\%}$ = instantaneous expiratory flow at 25% of FVC; V_T = tidal volume.
